# The Effect of Lumbar Belts with Different Extensibilities on Kinematic, Kinetic, and Muscle Activity of Sit-to-Stand Motions in Patients with Nonspecific Low Back Pain

**DOI:** 10.3390/jpm12101678

**Published:** 2022-10-09

**Authors:** Sang-Cheol Im, Seong-Wook Seo, Na-Yeon Kang, Hoon Jo, Kyoung Kim

**Affiliations:** Department of Physical Therapy, College of Rehabilitation Science, Daegu University, Gyeongsan 38453, Korea

**Keywords:** electromyography, kinematic, kinetic, low back pain, lumbar belt, sit-to-stand

## Abstract

Although lumbar belts can be used for the treatment and prevention of low back pain, the role of the lumbar belt remains unclear without clear guidelines. This study aimed to investigate the effect of lumbar belts with different extensibilities on the kinematics, kinetics, and muscle activity of sit-to-stand motions in terms of motor control in patients with nonspecific low back pain. A total of 30 subjects participated in the study: 15 patients with nonspecific low back pain and 15 healthy adults. Participants performed the sit-to-stand motion in random order of three conditions: no lumbar belt, wearing an extensible lumbar belt, and wearing a non-extensible lumbar belt. The sit-to-stand motion’s kinematic, kinetic, and muscle activity variables in each condition were measured using a three-dimensional motion analysis device, force plate, and surface electromyography. An interaction effect was found for the time taken, anterior pelvic tilt angle, and muscle activity of the vastus lateralis and biceps femoris. The two lumbar belts with different extensibilities had a positive effect on motor control in patients with nonspecific low back pain. Therefore, both types of extensible lumbar belts can be useful in the sit-to-stand motion, which is an important functional activity for patients with nonspecific low back pain.

## 1. Introduction

Low back pain is one of the most prevalent disorders today [1], and people with low back pain move differently than healthy people do [2]. In particular, changes in motion variability may indicate an altered adaptability of motor control systems [3,4]. As reported in previous studies, a decrease in motion variability during running [5] and bending [6] activities in patients with low back pain is a protective behavior when pain is present or when pain is expected [7], and repeated tissue stress can occur over time [8]. In addition, when the motion variability increases, as observed in reaching [9] and walking [10], in patients with low back pain, errors or noises in proprioceptive sensory feedback and motor commands may occur, thereby reducing the robustness of the system [11]. Thus, low back pain can cause significant functional loss and affect the performance of dynamic tasks and static postural control [12]. The sit-to-stand motion is a common and functionally important task in daily life activities [13]. Because it is associated with worsening low back pain symptoms, studies on the sit-to-stand motion are essential in back pain-related research [14].

Although there is hope that individualized treatment will be effective for people suffering from low back pain [15], most studies claiming to be effective have been shown to be flawed [16,17]. In addition, claims of treatment efficacy in subgroups of individuals with low back pain are reliable when there is a biological basis, but this is difficult in nonspecific low back pain [18]. Because nonspecific low back pain has no known pathological cause, treatment focuses on reducing pain and consists of education, analgesics, and psychotherapy, but many patients do not require medical treatment [19]. Strategies for preventing low back pain include ergonomic control at the workplace where repetitive movements and tasks are performed [20], exercises, such as training in waist mechanics and lifting techniques [21], and the use of lumbar belts, which can also be used to treat low back pain [22].

To explain the clinical benefits of lumbar belts, psychological, neuromuscular, and biomechanical mechanisms have been proposed, but these remain unproven [23]. Psychological benefits may be related to the perception of mechanical support generated by the lumbar belt, and neuromuscular benefits may include mechanisms that affect lumbar stability, such as lumbar proprioception, trunk muscle feedforward, and reflex activity [24,25]. Biomechanical benefits are related to the mechanical stiffness of the lumbar belt, such as a reduction in lumbar range of motion, a reduction in the stress of the posterior lumbar passive tissue, and a potential reduction in the compressive load of the lumbar spine [26,27]. However, the potential role of lumbar belts in treating low back pain remains unclear [28], and low-quality studies and insufficient evidence imply that there are no clear guidelines for using lumbar belts in patients with low back pain [29]. For this reason, it is necessary to study the effect of lumbar belts on the biomechanics and motor control mechanism of patients with low back pain.

Most previous studies on lumbar belt have analyzed proprioception or balance, and there is an insufficient number of studies on the effect of wearing a lumbar belt while the patient with low back pain performs a functional task. In particular, the effect of sit-to-stand motion, an important functional activity for modern people who spend a lot of time sitting and for patients with low back pain, has not been studied so far. Therefore, this study sought to investigate the effects of wearing two lumbar belts with different extensibilities on the kinematics, kinetics, and muscle activity in terms of motor control of the sit-to-stand motion in patients with nonspecific low back pain and healthy adults. From this research, we intend to present the evidence for the effective use of lumbar belts for patients with nonspecific low back pain and provide basic data necessary for the prevention and treatment of low back pain. For the purpose of this study, we hypothesized that the effects of the sit-to-stand motion on kinematics, kinetics, and muscle activity would differ depending on the presence or absence of back pain and the extensibility of the lumbar belt.

## 2. Methods

### 2.1. Participants

Participants in this study were recruited from outpatients and caregivers between the ages of 20 and 50 years who visited a spine specialty hospital located in Daegu, Korea, from April 2020 to May 2020. Of the 30 participants, 15 patients with nonspecific low back pain were assigned to the experimental group, and 15 normal adults were assigned to the control group. Participants in the control group were matched to those in the experimental group according to age, sex, and body mass index (BMI). We used G*Power software program (version 3.1.9.4, Franz Faul, Düsseldorf, Germany) to determine the appropriate number of subjects, and we referred to a previous study with the same design (two groups × three conditions) as this study [30]. We used the effect size of the lumbar total range of motion variable of the manual material-handling task in the previous study. Based on an effect size of 0.31, a significance level of 0.05, and a power of 80%, we calculated that a total of 30 subjects were required [31].

The selection criterion for the experimental group was lumbar or lumbar spine pain for at least 4 weeks without radiating pain below the knee (nonacute phase), and the exclusion criteria were spinal surgery, specific lumbar pathology, scoliosis, systemic or degenerative disease, BMI > 30 kg/m^2^, hypertension, neurological history unrelated to back pain, and medications that could affect neuronal excitability [30]. The exclusion criterion for the control group was a history of low back pain lasting more than 1 week during the past year [32]. Additional exclusion criteria for both groups were a neurological or respiratory disease, trauma, or pregnancy that could affect the sit-to-stand motion [33]. The orthopedic surgeon verified the qualifications of these participants through medical records and image evaluations and interviews.

After explaining the purpose of the study, the contents of the experiment, and the degree of exposure during the experiment to all participants in advance, written consent for voluntary participation was obtained. All participants’ sex, age, and anthropometric data were collected, and pain (visual analog scale) and disability (Oswestry Disability Index) data were additionally investigated for patients with nonspecific low back pain in the experimental group. This study was approved by the Bioethics Committee of Daegu University (1040621-201911-HR-025-02) and was conducted in accordance with the 1964 Declaration of Helsinki and its later amendments. In addition, this study was registered with the Clinical Research Information Service for information sharing, ethics, and transparency of clinical research (KCT0004970).

### 2.2. Lumbar Belts

After consulting with an orthopedic surgeon, two models of lumbar belt were selected, taking into consideration flexibility, comfort, and economy for use in daily life and work. These two types of lumbar belts consisted of two layers of straps secured with Velcro. The initial adjustment and placement of the lumbar belt were made with the inner layer, and the final tension was adjusted with the outer layer. The extensible lumbar belt (REDIX-K210, Acetech, Seoul, Korea) was made up of elastic materials, but the nonextensible lumbar belt (REDIX-L350, Acetech) was made up of nonstretchable nylon materials only. Ready-made products in three sizes (large, medium, and small) were used based on the body type of the participants. The position of the lumbar belt to be worn was such that the lower edge did not touch the thigh when sitting but covered the anterior superior iliac spine (ASIS) of the pelvis [32]. Then, while the participant was standing still, the wearing pressure was standardized to 60 mmHg as measured from a thin force-sensing resister sensor inserted between the waist belt and the participant’s right iliac crest [34].

### 2.3. Experimental Procedure

A comfortable environment was created so that participants did not feel uncomfortable with the temperature and surrounding environment of the laboratory. In order not to interfere with the measurement, all participants wore a sleeveless T-shirt made of lightweight material and short shorts. Before obtaining other measurements, body measurements were obtained by the same examiner to determine the participants’ general characteristics.

**Figure 1:**
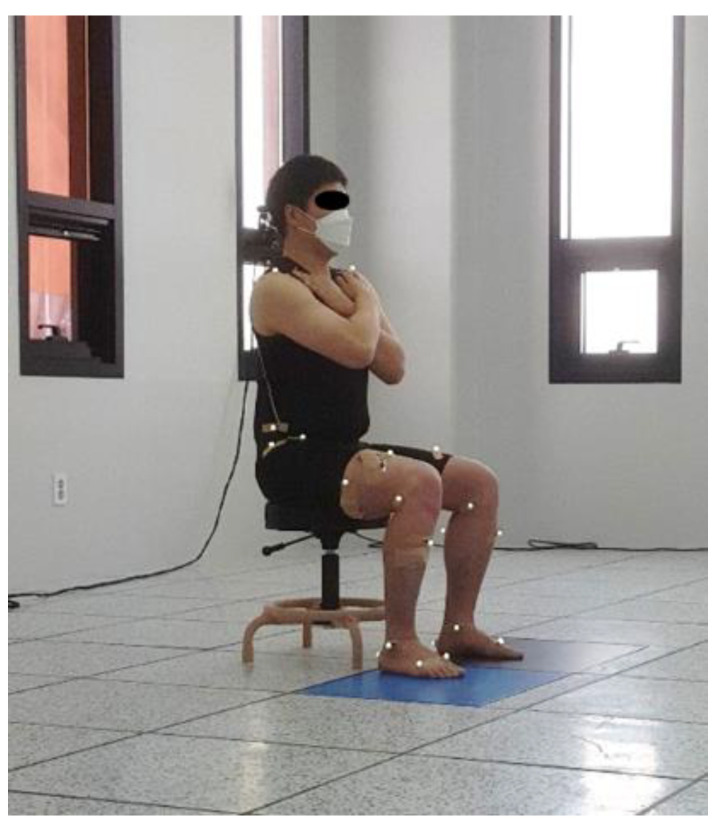
Experimental preparation posture.

When the electromyography (EMG) electrode placement and motion analysis reflex marker were prepared, the participants placed their bare feet at a comfortable width on two force plates installed on the floor. They sat down in height-adjustable chairs without armrests or backrests (Figure 1). The chair’s height was adjusted to be 90% of the length between the knee and the floor, which is the distance between the fibular head and the floor. Participants looked forward and sat in their preferred comfortable position, with their arms folded in front of their chest to avoid interference from shaking and occlusion of markers [35].

Following verbal cues from a research assistant operating a computer connected to the data acquisition system, the participants stood up at a rate of their choosing, held a comfortable upright position for 3 s, and then sat back down on their chairs without further instructions. To help the participants understand the experimental procedure, measurements were taken after two to three practice sessions following the researcher’s demonstration. Participants performed the sit-to-stand motion in the most comfortable way of their choosing, with the only limitation under which they did not change their feet positions. Paper tape was used to mark the initial position of the foot, and in the subsequent conditions, the sit-to-stand motion was performed with the same foot position.

**Figure 2:**
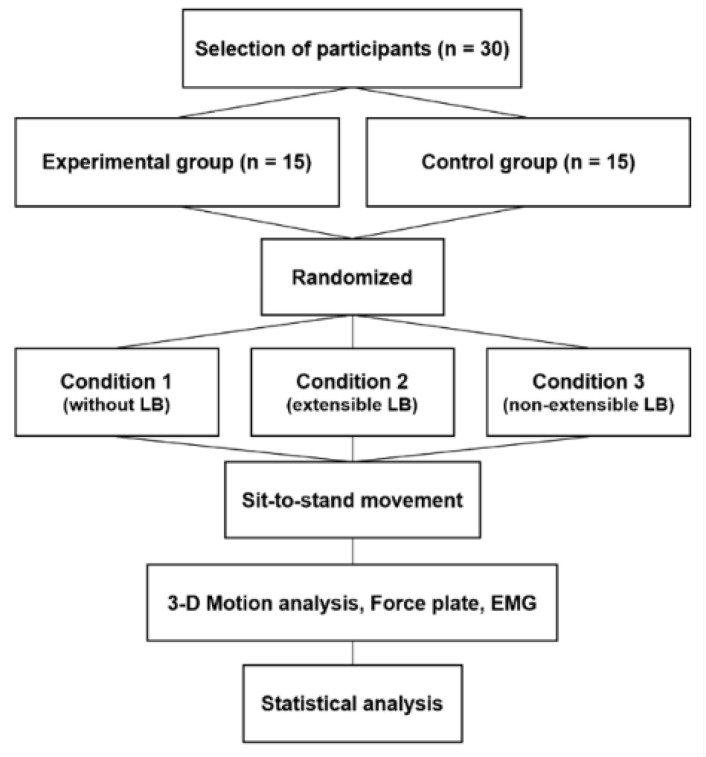
Study flowchart.

Three experimental conditions were set: no lumbar belt-wearing condition (condition 1), extensible lumbar belt-wearing condition (condition 2), and nonextensible lumbar belt-wearing condition (condition 3). To exclude a learning effect, the experimental conditions were conducted in a random order. A card with the experimental conditions was placed in a sealed envelope, and the experiment was conducted in the order in which each participant drew the card. The participants performed the sit-to-stand motion under each condition, and the biomechanical variables were then measured (Figure 2). To increase reliability, three successful measurements were taken for each condition, and the average value was used for analysis. All participants performed the sit-to-stand motion a total of nine times. The test was considered successful if all markers were visible and the EMG signals of all muscles were recorded correctly. There was a one-minute rest period between each test and a five-minute rest period between the lumbar belt conditions.

### 2.4. Measurement Method

#### 2.4.1. Measurement of Kinematic and Kinetic Parameters

In this study, we used a three-dimensional (3D) motion analysis system (Qualisys Medical AB, Gothenburg, Sweden) that included 6 infrared cameras and a force plate (Kistler, Winterthur, Switzerland) to collect the kinematic and kinetic data of the participants’ body movements when performing the sit-to-stand motion. The resolution of the Qualisys Motion Capture System was 1280 × 1024 pixels, and the sampling frequency was set to 200 Hz. The 3D spatial coordinates were made using a nonlinear transformation method. The sampling frequency of the force plate was set to 2000 Hz. All kinematic and kinetic data were measured using Qualisys Track Manager (Qualisys Medical AB) software and were low-pass filtered using a fourth-order Butterworth filter with cutoff frequencies of 25 Hz and 10 Hz. Data were then analyzed using Visual 3D software (C-Motion, Germantown, MD, USA) [36]. Moments were normalized by the system to weight and height [37].

Prior to conducting this test, we performed calibration to correct errors that may occur in the infrared camera. One examiner modified the methods of the previous study according to the purpose of this study and attached 36 reflective markers on the landmark skin of the body [38]. A static test was performed by attaching a reflective marker and checking the position of each joint on a computer screen in the still state of standing barefoot. During the static test, which was performed before measuring each experimental condition, the participant stood still for 2 s on the force plate. All markers were fixed by banding using Kinesio tape, which does not reflect light, to prevent shaking or falling. After the static examination, the length from both lateral ankles to the markers (ASIS, PSIS, top of iliac crest) that were covered when the lumbar belt was worn while standing upright and the length between each marker were recorded with a tape measure. In the lumbar belt–wearing condition, the marker was reattached by maintaining the same position between the experimental conditions using this length.

#### 2.4.2. Measurement of Muscle Activity

**Figure 3:**
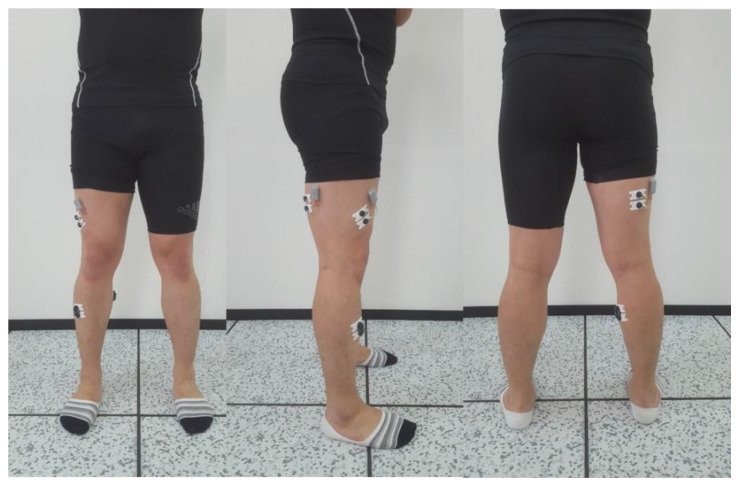
Attachment of EMG electrodes.

We measured the muscle activity of the vastus lateralis (VL), biceps femoris (BF), tibialis anterior (TA), and gastrocnemius (GM) muscles during the sit-to-stand motion using the Wireless Bipolar Cometa Mini Wave Plus EMG system (Cometa, Bareggio, Italy) synchronized with Visual 3D software (C-Motion). The sampling rate of the surface EMG signal was set to 2000 Hz. The raw surface EMG signal was rectified after band-pass filtering at 10–1000 Hz and low-pass filtered with a Butterworth filter [39]. As the ground electrode, a disposable anode surface electrode made of silver–silver chloride was used and attached parallel to the muscle fiber. The distance between electrodes in the surface EMG was set to 20 mm. To minimize the skin resistance and reduce the noise of muscle activity measurement, the hair on the skin was removed using a disposable razor, and after gently polishing with sandpaper, the skin was cleaned with alcohol. After the electrode attachment site was dried, two active electrodes and a reference electrode were attached. The electrodes of the four muscles of the dominant lower extremity (VL, BF, TA, GM) were placed according to the SENIAM guidelines [40] (Figure 3).

All of the measured raw EMG data were converted into root mean square, which provides a value close to the actual output value of the EMG signal. To analyze the activity of each muscle, the maximal voluntary isometric contraction (MVIC) of VL, BF, TA, and GM was measured, and the EMG signal collected during the sit-to-stand motion under each experimental condition was calculated as the percentage of MVIC [41]. Surface EMG data were synchronized using the pulses generated at the beginning of the recording of kinematics and kinetics, and the average muscle activity measurements during the sit-to-stand motion were compared.

### 2.5. Data Processing

**Figure 4:**
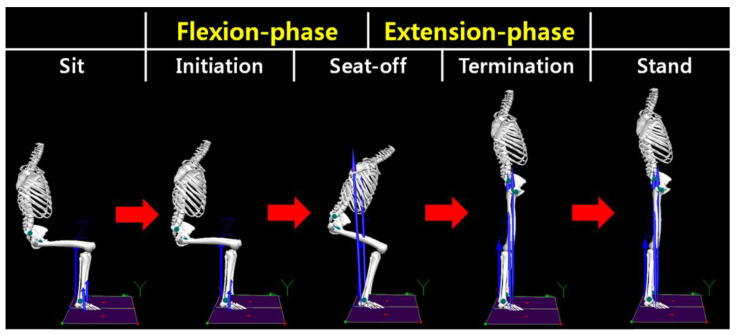
Indicators and classification of the sit-to-stand motion.

The sit-to-stand motion was divided into three points and two phases to conduct the analysis of the kinematics, kinetics, and muscle activity variables (Figure 4). The three points were determined using the ground reaction force (GRF) measured on the force plate. The initiation point [42] and the seat-off point [43] were determined based on the vertical GRF, and the termination point was determined based on the anterior–posterior GRF (A-P GRF) [44]. To detect potential indicator changes in the sit-to-stand motion, the minimum and maximum values of each GRF data were used for calculation. The initiation point was detected when a 5% threshold change occurred from the reference value of the vertical GRF in the sitting position, and the termination point was detected when a 7.5% threshold change occurred from the final value of A-P GRF in the standing position, going backward [45]. The seat-off point was set as the maximum value point of the vertical GRF [44]. The phase from the initiation point to the seat-off point was set as the flexion phase, and the phase between the seat-off point to the termination point was set as the extension phase [46]. Each sit-to-stand motion was normalized to 101 data points from the initiation point to the termination point [33]. The time from the initiation point to the termination point was measured, and the value of the seat-off point of the sit-to-stand motion was used for the angle of joints in the kinematic variable. The maximum value during the sit-to-stand motion was used for the moment in the kinetic variable. The average muscle activities of the extension and flexion phases were used for the muscle activity variable.

### 2.6. Statistical Analysis

For statistical processing of the data collected in this study, we used SPSS 22.0 for Windows (IBM Corp., Armonk, NY, USA), and all data are reported as mean ± standard deviation. The normality test was performed using the Shapiro–Wilk test. The participants’ general characteristics were analyzed using descriptive statistics, and an independent t test and a chi-square test were used to test the homogeneity of the two groups. Two-way repeated analysis of variance (ANOVA) was performed to confirm the interaction between the presence or absence of back pain and the change in lumbar belt-wearing conditions. If the interaction was significant, the Bonferroni method was used for post hoc analysis. The least square difference was used for the main effect comparison. When both the interaction and the main effect were significant, we analyzed the interaction only. The statistical significance level (α) was set to 0.05.

Cohen’s d formula was used for the effect size corresponding to the detected effect for the interaction between the experimental group and the control group, the main effect, and the comparison between groups [47]. The effect size was calculated using the G-power 3.1.9.4 program. In ANOVA, 0.10 was interpreted as “small,” 0.25 as “medium,” and 0.40 as “large” for effect size *f* [48].

## 3. Results

### 3.1. Analysis of The General Characteristics of Experimental and Control Groups

Table 1 shows the general characteristics of the experimental group and the control group. There was no significant difference between the two groups in terms of participants’ general characteristics (*p* > 0.05).

### 3.2. Analysis of the Time Taken, Kinematics, and Kinetics Variables According to Lumbar Belt Wearing Conditions of the Experimental Group and Control Group

There was an interaction effect in the required time (*p* < 0.05, *f* = 0.36; Table 2). As a result of the post hoc analysis, there was a significant difference between the condition in which the experimental group did not wear a lumbar belt (condition 1) and the conditions in which the lumbar belt was worn (conditions 2 and 3). There was an interaction effect in the pelvic anterior tilt angle (*p* < 0.05, *f* = 0.50; Table 2). Post hoc analysis showed a significant difference between the control group not wearing the lumbar belt (condition 1) and the lumbar belt–wearing conditions (conditions 2 and 3), and there was a significant difference between the control group and experimental group in the condition of not wearing the lumbar belt (condition 1).

There were main effects among lumbar belt–wearing conditions in trunk flexion angle, hip flexion angle, knee joint flexion angle, hip flexion–extension moment, and knee joint flexion–extension moment (*p* < 0.05, *f* = 0.36–0.75; Table 2). Post hoc analysis showed a significant difference between the condition in which the lumbar belt was not worn (condition 1) and the conditions in which the lumbar belt was worn (conditions 2 and 3). There was a main effect between groups in hip flexion–extension moment (*p* < 0.05, *f* = 0.65; Table 2). Post hoc analysis showed a significant difference between the control group and experimental group.

### 3.3. Analysis of Muscle Activity According to Lumbar Belt Wearing Conditions of the Experimental Group and Control Group

An interaction effect was observed in the VL flexion phase (*p* < 0.05, *f* = 0.52; Table 3). Post hoc analysis showed a significant difference between the control group and experimental group in the condition in which the lumbar belt was not worn (condition 1). There was an interaction effect observed in the BF flexion phase (*p* < 0.05, *f* = 0.46; Table 3). Post hoc analysis showed a significant difference between the condition in which the lumbar belt was not worn (condition 1) and the conditions in which the lumbar belt was worn (conditions 2 and 3) in the experimental group.

There was a main effect between the lumbar belt–wearing conditions in the BF extension phase, TA flexion phase, GM flexion phase, and GM extension phase (*p* < 0.05, *f* = 0.42–0.47; Table 3). Post hoc analysis showed a significant difference between the condition in which the lumbar belt was not worn (condition 1) and the conditions in which the lumbar belt was worn (conditions 2 and 3). There was a main effect between groups in VL extension phase and GM flexion phase (*p* < 0.05, *f* = 0.49–0.52; Table 3). The results of the post hoc analysis suggested there was a significant difference between the control group and experimental group.

## 4. Discussion

The purpose of this study was to investigate the effect of the presence or absence of back pain and the difference in the extensibility of the lumbar belt on kinematics, kinetics, and muscle activity in terms of motion control in the sit-to-stand motion. The results of this study indicated that there was an interaction effect according to the presence or absence of back pain and the difference in the extensibility of the lumbar belt, and the two types of lumbar belts with different extensibility had a similar effect overall.

### 4.1. Analysis of The Interaction between the Presence or Absence of Low Back Pain and the Extensibility of the Lumbar Belt

The interaction effect was found in the time taken to perform the sit-to-stand motion. The post hoc analysis indicated no difference between the lumbar belt–wearing conditions in the healthy adult group, but there was a decrease in the time taken in the patients with nonspecific low back pain in the conditions in which the lumbar belt was worn (conditions 2 and 3) compared with the condition in which the lumbar belt was not worn (condition 1). The lumbar belt had effects such as improving proprioception [49], increasing mechanical stiffness [23], and relieving pain [30]. Although the mechanical stiffness increased in both the nonspecific low back pain group and the healthy adult group, wearing a lumbar belt in this study reduced the required time only in the group with nonspecific low back pain. It has been reported that proprioceptive sensation is impaired in patients with low back pain [50,51], and impaired proprioceptive sensation is associated with motor control disorders [52]. Therefore, we believe that the required time was reduced because the lumbar belt improved the proprioceptive sense of the patients with nonspecific low back pain and had a positive effect on motor control. Considering the normal effect size, we believe that the lumbar belt was partially helpful in improving the sit-to-stand motion by reducing the time taken for the patients with nonspecific low back pain.

In the kinematics of the sit-to-stand motion, an interactive effect was found on the angle of pelvic anterior tilt at the seat-off point. Results of the post hoc analysis showed that the experimental group had a greater pelvic anterior tilt angle than the control group in the condition in which the lumbar belt was not worn (condition 1). In conjunction with the angle of trunk flexion in this experiment, this difference in the angle of anterior pelvic tilt is thought to be a protection strategy for patients with low back pain to reduce the angle of trunk flexion and maintain the lumbar region in a rigid state. In the control group, the angle of the anterior pelvic tilt was greater in the conditions in which the lumbar belt was worn (conditions 2 and 3) than in the condition in which the lumbar belt was not worn (condition 1). In a previous study analyzing the effect of the lumbar belt on the range of motion of the lumbar spine and the lumbar–pelvic rhythm, it was reported that wearing a lumbar belt restricts the range of motion of the lumbar spine and also changes the lumbar–pelvic rhythm [23]. In this study, the lumbar belt was worn so that the lower edge of the lumbar belt covered the ASIS and the iliac crest to include the pelvis, which may have had a stiffening effect on the lumbar belt, as in the previous study on the spine–pelvic connection. The forward-driving force that accelerates the body’s center of mass (COM) in the sit-to-stand motion is generated by trunk flexion [53]. In the experimental group, it is thought that the front driving force required to perform the sit-to-stand motion was generated as the trunk flexion angle increased by wearing the lumbar belt. In the control group, there was no change in the trunk flexion angle because the lumbar belt was worn, and the anterior pelvic tilt angle may have increased as a result of the stiffening effect of the lumbar belt. The interaction of the pelvic anterior tilt angle at the seat-off point showed a large effect size. Therefore, the difference in the angle of the anterior pelvic tilt is a characteristic that shows that the nonspecific low back pain patient group and the normal adult group performed the sit-to-stand motion differently, and the stiffening effect of the lumbar belt acting on the lumbar–pelvic connection is considered to be sufficient.

An interaction effect was found on the average muscle activity of VL and BF during the sit-to-stand motion. There was an interaction effect on the average muscle activity of the VL flexion phase. As a result of the post hoc analysis, the mean VL muscle activity of the nonspecific low back pain patient group was significantly higher than that of the healthy adult group under the condition in which the lumbar belt was not worn (condition 1). In this study, the nonspecific low back pain patient group showed a tendency to perform the sit-to-stand motion using the knee joint, increasing the angle and extension moment of the knee joint while keeping the lumbar–pelvic region in a rigid state. Thus, we believe that VL was involved in knee joint control in the flexion phase, and muscle activity was high in the patients with nonspecific low back pain. A previous study also reported that the increase in the knee joint extension moment was due to the high activity of the knee joint extensors, which supports the results of this study [54]. There was an interaction effect on the average muscle activity of the BF flexion phase. As a result of the post hoc analysis, there was no difference between the lumbar belt–wearing conditions in the healthy adult group; however, as compared with the condition without the lumbar belt (condition 1), the average muscle activity was significantly decreased in the nonspecific low back pain patient group in the condition with the lumbar belt (conditions 2 and 3). According to a study using EMG, the compensatory mechanism of muscle activation in the painful state could occur in antagonist muscles and induce changes in the pattern of muscle recruitment [55]. Because the sit-to-stand motion is an action that causes the body to stand up against gravity, the knee joint is regulated by the contraction of VL in the flexion phase. Therefore, the high muscle activity of the BF, the antagonist of the VL, in the condition that the lumbar belt is not worn (condition 1) is a change in the muscle recruitment pattern and is considered to be a compensatory mechanism in patients with nonspecific low back pain. According to the results of this study, the muscle activity of the VL and BF was reduced as the compensatory mechanism was reduced because the trunk flexion angle of the nonspecific low back pain patient group was increased by wearing a lumbar belt, which secured the trunk flexion angle necessary for the sit-to-stand motion. We found a large effect size of the interaction in the muscle activity of the VL and BF. From this large effect size, the lumbar belt is considered to have an advantage in terms of motor control.

### 4.2. Analysis of Main Effects between Patients with Nonspecific Low Back Pain and Healthy Adults

During the sit-to-stand motion, there was a difference between the nonspecific low back pain patient group and the healthy adult group. The extension moment of the healthy adult group was significantly greater in the flexion–extension moment of the hip joint. Although the extension moment of the nonspecific low back pain patient group was not statistically significant in the flexion–extension moment of the knee joint, it tended to be larger. Healthy individuals complete the process of standing upright by simultaneously flexing the spine and hip and then simultaneously extending the spine and hip when performing a sit-to-stand motion [56]. In this study, the healthy adult group also correctly performed the sit-to-stand motion using the hip extension moment. In contrast, in the nonspecific low back pain patient group, the sit-to-stand motion was performed using the knee joint rather than the hip joint. The surface EMG results of the lower extremity muscles showed a tendency for the muscle activity to be high in the nonspecific low back pain patient group. In the flexion phase, the VL and GM muscle activity of the nonspecific low back pain patient group was high, and in the extension phase, the VL muscle activity of the nonspecific low back pain patient group was high. In a previous study, the authors claimed there was a causal relationship between motor outcome and neuromuscular activity to create a protective mechanism [2]. In the flexion phase, the VL and GM play a role in regulating the knee and ankle joints, and in the extension phase, the VL more greatly extends the flexed knee joint; thus, it is considered that the muscle activity of the nonspecific low back pain patient group was high.

Previous studies have discussed the various mechanisms for nonspecific low back pain [57], and motor control disorders have been reported to possibly be one of the causes [58]. Because of the link between neuromuscular activity and biomechanical consequences, changes in the neuromuscular system resulting from motor control disorders lead to slower movement and affect the movement of higher muscle activity [59]. In this study, the nonspecific low back pain patient group performed the sit-to-stand motion with the lumbar spine rigid by increasing the angle of pelvic anterior tilt and decreasing the angle of trunk flexion. This could be due to a short-term protective strategy to reduce the load on the posterior structure of the lumbar spine and avoid further stress on the painful area. In addition, to compensate for reduced body flexion angle, the knee joint flexion angle was increased when performing the sit-to-stand motion. This is consistent with the results of the nonspecific low back pain patient group, which had a small hip extension moment and a large knee joint extension moment, as compared with the healthy adult group. Previous studies also reported that in patients with low back pain, the lower extremities were involved as a possible compensatory exercise for pain [60]. As such, we confirmed that movement control disorders in patients with nonspecific low back pain had an effect on daily activities, such as performing the sit-to-stand motion.

### 4.3. Analysis of The Main Effects between Lumbar Belt Wearing Conditions

The trunk flexion angle, hip flexion angle, and hip joint extension moment at the seat-off point of the sit-to-stand motion significantly increased in the conditions with the lumbar belt (conditions 2 and 3) compared with the condition without the lumbar belt (condition 1). In the knee joint, flexion angle and extension moment were significantly decreased. The lumbar belt can reduce the stress on the posterior viscoelastic structure of the lumbar vertebrae or the compression load of the lumbar vertebrae [26,27]. This is an effect of the limited lumbar movement due to the increased mechanical rigidity when the lumbar belt is worn, and it can prevent the load of a specific vertebral structure [61]. In this study, we found that the protection mechanism for reducing the angle of trunk flexion was reduced by wearing a lumbar belt to avoid stress on the painful area and thus the angle of trunk flexion was increased. Trunk flexion in the sit-to-stand motion creates a forward-driving force that accelerates the body’s COM [53]. We believe that the compensatory action occurring at the knee joint was reduced because the angle of trunk flexion and hip flexion increased by wearing the lumbar belt.

The EMG results showed that by wearing the lumbar belt, the muscle activity in the flexion phase significantly decreased in the TA, and the muscle activity in the flexion phase and extension phase in the GM significantly decreased. The TA and GM are involved in the control of knee and ankle joints in the sit-to-stand motion. Previous studies also reported that the TA prepares for anterior displacement of the body’s COM by moving the tibia forward [62]. It is thought that the muscle activity of the TA and GM decreased as the knee joint flexion angle decreased as a result of lumbar belt wearing. There was a significant increase in the muscle activity in the extension phase of the BF. Compensation is a motor strategy used to restore postural balance by sensory feedback [63]. In a previous study, a relatively small activity of the hip extensor was reported because small trunk flexion required less forward speed of the body’s COM [54]. In this study, we consider that the muscle activity of the BF acting as a hip extensor increased because the body flexion angle increased by wearing a lumbar belt, which required greater forward speed of the body’s COM.

### 4.4. Limitations

Because of the relatively small number of subjects in this study, it is difficult to generalize the results. We investigated only the immediate effect of the lumbar belt as opposed to the long-term effect. Because the spine was investigated as a single body, the movement of each vertebral segment and the compensatory action of the thoracic vertebrae were not considered. To focus on the effect of the lumbar belt on the sit-to-stand motion, there were restrictions on the height of the chair and the position of the feet. Therefore, different results may be obtained if the chair height and foot position are freely set. In addition, because the types and designs of the lumbar belts are diverse, different results could be obtained if there is a difference in the extensibility and design of the lumbar belt used. Finally, the muscle activity of the trunk muscles could not be measured because it could be disturbed by wearing a lumbar belt. Therefore, further studies using more diverse lumbar belt designs, including study designs using more subjects and a placebo effect, and lumbar belts equipped with biofeedback devices are needed in the future.

## 5. Conclusions

Patients with nonspecific low back pain performed the sit-to-stand motion in a pattern different from that of healthy adults, resulting in impaired motor control and decreased ability. The two lumbar belts with different extensibilities had a positive effect on motor control and improved performance in patients with nonspecific low back pain. Therefore, both types of lumbar belts with different extensibilities can help with the sit-to-stand motion, which is an important functional activity for patients with low back pain.

## Figures and Tables

**Table 1:** General characteristics of the experimental and control groups.

Variable	CG (*n* = 15)	EG (*n* = 15)	*p*
Sex (Male, %)	9 (60.0)	8 (53.3)	0.500 ^†^
Age (year)	34.53 (4.40)	35.60 (5.19)	0.549 ^‡^
Height (cm)	171.40 (9.83)	170.20 (8.08)	0.718 ^‡^
Weight (kg)	70.60 (12.50)	67.40 (14.60)	0.525 ^‡^
BMI (kg/m^2^)	23.85 (2.26)	23.02 (3.38)	0.437 ^‡^
Visual analog scale (score)		3.80 (0.86)	
Oswestry Disability Index (%)		19.60 (4.42)	
Onset (month)		43.06 (33.35)	

^†^ Chi-squared test, ^‡^ Independent *t*-test, CG: Control group, EG: Experimental group.

**Table 2:** Analysis of the time taken, kinematics, and kinetics variables for the sit-to-stand motion according to the experimental conditions.

Variable	Condition 1 (No LB)		Condition 2 (Extensible LB)		Condition 3 (Nonextensible LB)		*p* (*f*)			Post Hoc
	CG	EG	CG	EG	CG	EG	“Group” (G)	“Condition” (C)	G × C	
Time taken (s)	1.76 (0.19)	1.90 (0.28)	1.76 (0.22)	1.78 (0.20)	1.75 (0.22)	1.77 (0.20)	0.401	0.018 * (0.39)	0.033 * (0.36)	EG: C1 > C2, C1 > C3
Trunk flexion angle (°)	45.70 (10.42)	40.79 (6.41)	45.96 (10.07)	45.17 (6.12)	46.89 (10.28)	44.09 (5.65)	0.336	0.030 * (0.38)	0.102	C1 < C2, C1 < C3
Pelvic anterior tilt angle (°)	34.14 (5.94)	42.05 (6.16)	45.08 (8.30)	45.46 (8.89)	45.55 (8.57)	44.74 (6.31)	0.290	<0.001 * (0.87)	0.002 * (0.50)	CG: C1 < C2, C1 < C3 C1: CG < EG
Hip flexion angle (°)	100.16 (8.82)	104.30 (11.45)	108.07 (13.42)	110.73 (10.79)	111.97 (11.86)	110.24 (9.72)	0.640	<0.001 * (0.75)	0.196	C1 < C2, C1 < C3
Knee flexion angle (°)	73.13 (9.09)	77.05 (9.34)	72.13 (7.82)	74.77 (9.29)	71.77 (8.94)	75.08 (9.13)	0.308	0.028 * (0.37)	0.655	C1 > C2, C1 > C3
Ankle flexion angle (°)	−6.74 (4.28)	−5.73 (3.71)	−6.14 (4.91)	−6.61 (3.85)	−6.41 (4.44)	−6.57 (5.92)	0.933	0.908	0.422	
Hip flexion–extension moment (nm/kg)	−1082.71 (272.10)	−875.20 (171.62)	−1163.58 (213.83)	−968.21 (112.71)	−1193.42 (222.59)	−948.38 (151.12)	0.002 * (0.65)	0.005 * (0.45)	0.699	CG > EG C1 < C2, C1 < C3
Hip adduction–abduction moment (nm/kg)	202.97 (164.75)	178.82 (139.13)	215.26 (117.89)	181.27 (103.57)	203.81 (94.11)	186.31 (88.26)	0.532	0.848	0.821	
Hip Int. Rot–Ext. Rot moment (nm/kg)	176.89 (117.37)	170.56 (103.88)	177.02 (163.95)	159.37 (112.66)	185.39 (186.78)	188.46 (109.36)	0.883	0.365	0.675	
Knee flexion-extension moment (nm/kg)	−593.82 (145.11)	−702.46 (168.38)	−563.46 (153.38)	−655.45 (161.79)	−549.03 (134.16)	−661.88 (155.61)	0.055	0.033 * (0.36)	0.820	C1 > C2, C1 > C3
Knee varus–valgus moment (nm/kg)	−70.03 (32.95)	−70.54 (57.46)	−70.41 (38.22)	−73.85 (56.54)	−60.15 (43.77)	−61.66 (57.35)	0.912	0.158	0.971	
Knee Int. Rot–Ext. Rot moment (nm/kg)	−108.82 (62.90)	−108.31 (67.98)	−110.77 (64.01)	−107.18 (76.34)	−102.12 (55.16)	−111.63 (68.13)	0.934	0.972	0.763	
Ankle flexion–extension moment (nm/kg)	107.93 (43.72)	104.49 (34.09)	113.31 (34.75)	96.59 (37.56)	108.47 (52.49)	93.34 (30.89)	0.322	0.756	0.621	
Ankle inversion–eversion moment (nm/kg)	29.02 (25.26)	30.87 (21.77)	32.93 (26.19)	34.26 (25.48)	30.96 (24.01)	36.06 (22.46)	0.750	0.091	0.551	
Ankle adduction–abduction moment (nm/kg)	−1.54 (6.61)	−4.37 (7.25)	−2.36 (9.06)	−3.12 (4.34)	−3.76 (7.41)	−2.69 (6.91)	0.708	0.912	0.243	

* *p* < 0.05, *f*: effect size “f”, CG: Control group, EG: Experimental group, C1: Condition 1, C2: Condition 2, C3: Condition 3.

**Table 3:** Analysis of muscle activity variables of the sit-to-stand motion according to the experimental conditions (%MVIC).

Variable	Condition 1 (No LB)		Condition 2 (Extensible LB)		Condition 3 (Nonextensible LB)		*p* (*f*)			Post Hoc
	CG	EG	CG	EG	CG	EG	“Group” (G)	“Condition” (C)	G × C	
Vastus lateralis flexion phase	18.16 (6.48)	31.71 (10.52)	21.00 (7.14)	28.28 (9.04)	20.39 (7.97)	28.35 (9.86)	0.003 * (0.61)	0.762	0.003 * (0.52)	C1: CG < EG
Vastus lateralis extension phase	28.50 (12.09)	40.89 (13.92)	27.41 (12.94)	40.39 (14.48)	29.59 (11.53)	41.04 (13.94)	0.014 * (0.49)	0.342	0.728	CG < EG
Biceps femoris flexion phase	3.59 (1.44)	5.05 (2.87)	3.96 (1.41)	4.47 (2.38)	3.85 (1.37)	4.23 (2.87)	0.312	0.277	0.004 * (0.46)	EG: C1 > C2, C1 > C3
Biceps femoris extension phase	9.37 (4.39)	8.09 (4.63)	9.94 (4.40)	10.11 (4.92)	10.28 (5.25)	10.68 (6.69)	0.893	0.007 * (0.47)	0.219	C1 < C2, C1 < C3
Tibialis anterior flexion phase	19.91 (13.06)	24.47 (14.29)	17.97 (8.95)	21.58 (11.68)	15.49 (8.77)	22.19 (13.59)	0.246	0.007 * (0.44)	0.321	C1 > C2, C1 > C3
Tibialis anterior extension phase	5.31 (3.17)	9.19 (4.92)	5.06 (3.37)	8.14 (6.86)	5.91 (3.44)	7.78 (6.22)	0.092	0.423	0.136	
Gastrocnemius flexion phase	3.08 (1.42)	4.26 (1.75)	2.75 (1.39)	3.83 (1.48)	2.24 (1.03)	4.06 (1.56)	0.010 * (0.52)	0.011 * (0.42)	0.076	CG < EG C1 > C2, C1 > C3
Gastrocnemius extension phase	5.25 (2.32)	6.65 (2.67)	4.76 (1.84)	5.68 (1.76)	3.91 (1.80)	5.59 (2.55)	0.063	0.005 * (0.45)	0.565	C1 > C2, C1 > C3

* *p* < 0.05, *f*: effect size “f”, CG: Control group, EG: Experimental group, C1: Condition 1, C2: Condition 2, C3: Condition 3.

## Data Availability

The data presented in this study are available on request from the corresponding author. The data are not publicly available due to ethical restrictions.
